# Ion and Water Transport in Neutrophil Granulocytes and Its Impairment during Sepsis

**DOI:** 10.3390/ijms22041699

**Published:** 2021-02-08

**Authors:** David Alexander Christian Messerer, Hanna Schmidt, Manfred Frick, Markus Huber-Lang

**Affiliations:** 1 Institute of Clinical and Experimental Trauma-Immunology, University Hospital of Ulm, 89081 Ulm, Germany; markus.huber-lang@uniklinik-ulm.de; 2 Department of Anesthesiology and Intensive Care Medicine, University Hospital of Ulm, 89081 Ulm, Germany; 3 Institute of General Physiology, Ulm University, 89081 Ulm, Germany; hanna.schmidt@uni-ulm.de (H.S.); manfred.frick@uni-ulm.de (M.F.); 4 Department of Pediatrics and Adolescent Medicine, University Hospital of Ulm, 89081 Ulm, Germany

**Keywords:** neutrophil granulocytes, calcium, NADPH oxidase, intracellular pH, chemotaxis, cell death, sepsis, NHE1

## Abstract

Neutrophil granulocytes are the vanguard of innate immunity in response to numerous pathogens. Their activity drives the clearance of microbe- and damage-associated molecular patterns, thereby contributing substantially to the resolution of inflammation. However, excessive stimulation during sepsis leads to cellular unresponsiveness, immunological dysfunction, bacterial expansion, and subsequent multiple organ dysfunction. During the short lifespan of neutrophils, they can become significantly activated by complement factors, cytokines, and other inflammatory mediators. Following stimulation, the cells respond with a defined (electro-)physiological pattern, including depolarization, calcium influx, and alkalization as well as with increased metabolic activity and polarization of the actin cytoskeleton. Activity of ion transport proteins and aquaporins is critical for multiple cellular functions of innate immune cells, including chemotaxis, generation of reactive oxygen species, and phagocytosis of both pathogens and tissue debris. In this review, we first describe the ion transport proteins and aquaporins involved in the neutrophil ion–water fluxes in response to chemoattractants. We then relate ion and water flux to cellular functions with a focus on danger sensing, chemotaxis, phagocytosis, and oxidative burst and approach the role of altered ion transport protein expression and activity in impaired cellular functions and cell death during systemic inflammation as in sepsis.

## 1. Introduction

“Water is the driving force in nature”; this quote attributed to Leonardo da Vinci (1452–1519), the Italian Renaissance man, indicates that water is crucial for life. Input and output of water and ions, therefore, require tight regulation in organisms. On a cellular level, various ion and water transport proteins ensure the ion–water balance, which is involved in any physiological and immune function.

A major function of the innate immune system is to supply the “first line” of the fluid and cellular defense system, comprising mainly the complement system and neutrophil granulocytes (neutrophils). The complement cascade becomes activated by numerous microorganism and microbial-associated molecular patterns (MAMPs) and in turn generates anaphylatoxins, opsonins, and membrane attack complexes, all of which support bacterial marking and clearance [1,2]. Neutrophils kill bacteria by a rather dynamic process, including pathogen sensing, targeted migration, phagocytosis, formation of an oxidative burst, generation of proteases, and formation of neutrophil extracellular traps (NETs), all of which finally help to control invading pathogens [3,4]. However, when the infectious agents outnumber the host’s defense capacities, or when the microorganisms are too virulent, or the host is somehow immune compromised, neutrophils and their associates, macrophages, may become overwhelmingly activated and finally dysfunctional in their function. Such a scenario can occur during the systemic inflammatory response in sepsis [3,5,6,7]. Recently, this neutrophil dysfunction has been increasingly associated with alterations in cellular size and intracellular cytosolic pH (henceforth referred to as “intracellular pH”), making an involvement of an ion–water disbalance likely [8,9,10,11,12,13,14,15,16].

Sepsis is defined as a life-threatening organ dysfunction caused by a dysregulated host response to infectious microorganisms and corresponding MAMPs [17]. With an annual incidence of approximately 50 million, sepsis-related deaths are estimated to represent almost 20% of all deaths globally [18]. Clinical symptoms consist of impaired consciousness, lowered blood pressure, and enhanced respiratory rate. Sepsis is frequently associated with an occult or evident edema. The underlying swelling of the interstitium and cells of various tissues is eventually induced by altered ion and water transporters [14]. These changes during sepsis may occur not only in solid organs, including the kidneys and lungs, where they enhance the distances of the blood-organ barrier with subsequent functional problems, but also in the cellular line of defense [7,11]. Therefore, changes in the ion–water homeostasis triggered by excessive inflammation contribute to immune and organ dysfunctions [4,5,10,14].

In this review, we discuss neutrophil functions, with a focus on ion and water flux with an emphasis on danger sensing, chemotaxis, phagocytosis, and oxidative burst, highlight pyroptosis and NETosis as neutrophil-related forms of cell death, and transfer these findings into the clinically relevant context of sepsis.

## 2. Extra- and Intracellular Danger Sensing

Danger sensing of harmful MAMPs, but also of damage-associated molecular patterns (DAMPs), is crucial for any organism, if not a “question of life and death”. Neutrophils, as the most frequent representatives of the first cellular line of defense, can recognize molecular danger by pattern recognition receptors (PRPs), including the membrane-bound formyl-peptide receptors (FPRs), toll-like receptors (TLR; e.g., TLR2 and TLR4), complement receptors (e.g., CR1, CR3, C5aR1/2), and receptor for advanced glycation end products (RAGE) [4,19,20]. There is also an effective arsenal of intracellular PRRs, such as TLR3, TLR7, TLR8, and TLR9, which are located in the endoplasmic reticulum. Further intracellular defense is provided by cytoplasmic nucleotide-binding oligomerization domains (NODs), e.g., NOD1, which recognizes a peptidoglycan-related molecules from most gram-negative and certain gram-positive bacteria, and NOD2, which senses muramyl dipeptides of all gram-positive and gram-negative bacteria [21]. The consequences of danger sensing during sepsis are omnifarious. On the one hand, MAMPs and DAMPs sensing via TLRs is an important pathway for neutrophils to recognize and clear invading bacteria [22,23]. On the other hand, DAMPs can compromise neutrophil functions. For example, high mobility group box 1 (HMGB1) is released and functions as both DAMP and inflammatory factor during both sterile and infectious inflammation, binds to RAGE and TLR4 on neutrophils, resulting in impairment of nicotinamide adenine dinucleotide phosphate oxidase (NOX) activation and bacterial killing. This can exacerbate sepsis-related organ dysfunction and outcome [5,6,24]. In the context of thromboinflammation and sepsis, an indirect mechanism for pathogen sensing has been proposed, whereby platelets can efficiently sense pathogens via various membrane-bound receptors (e.g., TLR4), and then secrete various platelet-associated molecules such as thrombospondin or platelet-activating factor (PAF), which can interact with neutrophils and other immune cells and thus transmit the MAMP or danger signal to neutrophils [25,26].

Multiple mechanisms exist by which microbes or corresponding MAMPs can enter host cells. For example, lipopolysaccharide (LPS) can enter the cytoplasm by escaping the bacteria/MAMP-containing vacuole, or via endocytic uptake of LPS encapsulated in outer membrane vesicles originated from bacteria [27]. Upon intracellular MAMP exposure, the innate immune sensor protein NOD-like receptor (NLR) family pyrin domain containing 3 (NLRP3) forms the inflammasome to cleave caspase-1, which in turn leads to interleukin (IL)-1β generation [28]. Recent studies on macrophages indicated that chloride intracellular channels (CLIC), a class of highly conserved but rather unconventional ion channels [29,30], which induce a Cl^−^ efflux, promote NLRP3 inflammasome assembly and subsequent IL-1β secretion, and thus mediate a proinflammatory response [31,32]. Whether the intracellular chloride homeostasis also alters the intracellular sensing and inflammatory response in neutrophils is currently unknown. It is noteworthy that MAMPs from bacteria, like LPS, can themselves modulate the sensing mechanisms and thereby impair subsequent vital neutrophil function as an immune evasion strategy [20].

Sensing of *N*-formylmethionine-containing oligopeptides (which are released by many invading microorganisms) via FPRs on neutrophils results in an increase in the mitochondrial membrane potential. This rapidly leads to a robust release of adenosine triphosphate (ATP) by the neutrophil. In turn, the excreted ATP subsequently activates purinergic receptors on the cellular surface in an autocrine manner. The resulting Ca^2+^-influx finally triggers functional changes, including morphological polarization, directed chemotaxis, phagocytosis, and degranulation [33,34].

## 3. Directed Chemotactic Activity

Cellular migration is a key function of neutrophils, which is induced by MAMPs from the inflammatory/infectious site (e.g., by *N*-formyl-methionyl-leucyl-phenylalanine (fMLF, previously termed fMLP)) and numerous inflammatory mediators, including among others complement factor 5a (C5a), IL-8, lipid mediators, such as PAF and leukotriene B_4_ (LTB_4_), and ATP [1,4,35,36,37].

For directional sensing of extracellular danger and the persistent directional migration towards a chemokine or C5a gradient, the cell division control protein 42 (Cdc42), a small GTPase of the Rho family, is required in neutrophils [38,39]. Chemotactic movement is evidently dependent on actin polymerization and cytoskeletal remodeling processes as well as osmotic conditions for the cell shape and size, respectively. For example, Cdc42 in neutrophils is not only activated by C5a but also by osmotic stress, resulting in actin polymerization and remodeling as well as subsequent shape changes [11,40,41]. In addition, acidosis as a sepsis-associated change of the local extracellular microenvironment, altered the chemotactic features towards enhanced sensitivity and decreased directionality to an fMLF gradient. In the case of an intracellular acidotic microenvironment, the chemotactic migratory activity of neutrophils towards fMLF was pH dependently reduced [42,43]. Taken together, there is a gap in the research on the association of ion and water transport (patho-)physiology with danger sensing and immunological consequences of neutrophils, particularly in the context of sepsis.

## 4. Sequential Neutrophil Activation after Chemoattractant Stimulation

**Figure 1:**
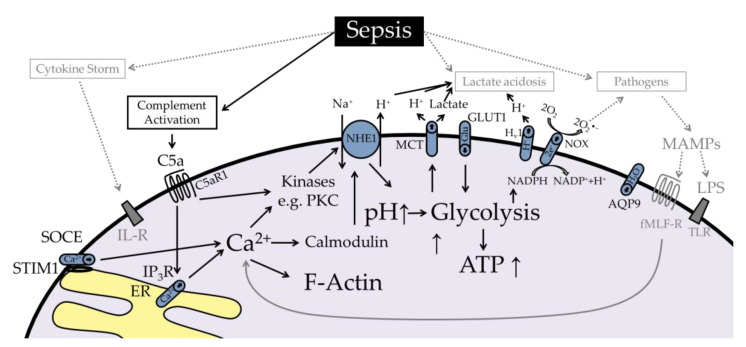
Simplified summary of the response of neutrophils upon stimulation with chemoattracttants with a focus on the well-studied C5a-C5aR1 axis. The anaphylatoxin C5a induces an increase in intracellular Ca^2+^, depolarization, and changes in cellular pH and size. fMLF induces similar signaling. AQP9-regulation has hitherto only been demonstrated for fMLF. SOCE = store-operated calcium entry, STIM1 = stromal interaction molecule 1, ER = endoplasmic reticulum, IP_3_R = inositol 1,4,5-trisphosphate receptor, IL-R = interleukin receptor, C5a = complement factor 5a, C5aR1 = C5a receptor 1, AQP9 = aquaporin 9, CaM = calmodulin, PKC = protein kinase C, NHE1 = sodium-proton exchanger 1, MCT = monocarboxylate transporter, Glu = glucose, GLUT1 = glucose transporter 1, NOX = NADPH oxidase, H_v_1 = voltage-sensing domain only protein, MAMP = microbial-associated molecular pattern, fMLF = *N*-formylmethionyl-leucyl-phenylalanine, fMLF-R = fMLF receptor, TLR = toll-like receptor.

In general, neutrophils respond to exposure to chemoattractants with a defined reaction pattern consisting of a rise in cytosolic Ca^2+^ [44,45,46,47] and depolarization of the membrane potential within seconds [44,45,46,48] as well as intracellular alkalization and increased cellular size within minutes (Figure 1) [10,11,42,49,50]. In this context, alterations in the response of neutrophils after chemoattractant stimulation are a hallmark of cellular dysfunction during sepsis.

### 4.1. Calcium Signaling

Upon stimulation, neutrophils respond with a rapid and pronounced increase in cellular Ca^2+^ levels [44,45,46,47,51,52]. Ca^2+^ signaling can be initiated by G protein-coupled receptors (GPCRs, e.g., receptors for fMLF or C5a) or in general by other ligand-receptor interactions, including Fcɣ-receptors (antibodies), and the activation of CD11b/CD18 (β2-integrin, e.g., by iC3b or fibrinogen) as well as by cellular crosstalk, such as platelet–neutrophil interaction (P-selectin glycoprotein ligand-1 and P-Selectin as well as CD40 and CD40L, respectively) [35,47]. GPCR activation results in the dissociation of the subunits of the G protein, which activate members of the phospholipase β and ɣ families, resulting in the cleavage of phosphatidylinositol 4,5-bisphosphate (PIP_2_) into inositol 1,4,5-trisphosphate (IP_3_), and diacylglycerol (DAG) [47,52,53,54]. IP_3_ triggers Ca^2+^ release from the endoplasmic reticulum (ER), causing an initial rise in intracellular Ca^2+^. The decrease in Ca^2+^ concentration in the ER induces store-operated calcium entry (SOCE). Stromal interaction molecule (STIM) 1 and 2 on the ER membrane translocate to defined regions at the plasma membrane, interacting with Ca^2+^ release-activated Ca^2+^ (CRAC) channels (predominantly Orai1 in neutrophils) to initiate the influx of extracellular Ca^2+^ [47,52,55,56,57]. In addition to SOCE-dependent mechanisms, CD38 (cyclic adenosine diphosphate (ADP) ribose hydrolase) adds to the elevation in intracellular Ca^2+^ of fMLF-stimulated neutrophils by the generation of the nicotinamide adenine dinucleotide (NAD^+^) metabolites ADP-ribose (ADPr) and to a lesser extent cyclic ADP-ribose (cADPr) [58], which in turn open transient receptor potential melastatin 2 (TRPM2) (extracellular Na^+^/Ca^2+^-influx) [47,59] and ryanodine receptors-gated stores (Ca^2+^-influx from the ER, independent of IP_3_-SOCE Ca^2+^-stores) [51,60,61], respectively. Ultimately, the rise in intracellular Ca^2+^ activates several kinases as well as ion channels and cellular functions, as reviewed in [47] and [52]. For example, the increase in Ca^2+^ activates various isoforms of protein kinase C (PKC), which phosphorylate among others p47 phagocyte oxidase (p47^phox^, synonym: neutrophil cytosolic factor 1), an important step in the activation of the multicomponent enzyme NOX [62,63,64,65]. In addition, the rapid increase in intracellular Ca^2+^ triggers secondary (electro-)physiological changes, including cellular depolarization via NOX activation [52,66,67,68], intracellular alkalization [10], and cellular shape changes [11], as discussed below.

### 4.2. Electron and Proton Fluxes Mediate Depolarization and Intracellular Alkalization

Neutrophil stimulation results in the activation of NOX, which transfers two electrons from nicotinamide adenine dinucleotide phosphate (NADPH) to molecular oxygen (O_2_), thereby generating superoxide anions (O_2_^•−^) [64]. In this process, electrons are transported across the plasma membrane (or into the phagolysosome), which induces cellular depolarization [45,64,69,70,71]. This depolarization must be compensated, because it otherwise limits the functionality of the NOX-mediated electron export and affects Ca^2+^ signaling [64,68,72]. To counterbalance this efflux of negative charge, neutrophil depolarization is accompanied by a H^+^ efflux via voltage-gated proton channels (voltage sensing domain only protein (VSOP) / H_v_1 as its respective human homologue) [64,68,73]. The striking relevance of this mechanism was further demonstrated in H_v_1-deficient mice, which responded to stimulation by the chemoattractant fMIVIL (*N*-formyl-Met-Ile-Val-Ile-Leu, stimulant of the murine equivalent of the fMLF receptor) with cellular acidification as well as a significantly reduced rise in Ca^2+^ and subsequent impaired actin depolymerization and cellular migration [68]. Likewise, neutrophils from patients with chronic granulomatous disease (pathology including several defects in NOX assembly) responded with almost no detectable depolarization under fMLF stimulation [46].

The rise in intracellular Ca^2+^ as well as the intracellular accumulation of H^+^ generated by NOX initiate H^+^ extrusion, via H_v_1 and the sodium-proton exchanger 1 (NHE1) [10,74,75,76,77]. H_v_1 is a voltage-dependent H^+^ channel activated by cellular depolarization [64,72,78]. The stimulation by chemoattractants and subsequent increase in intracellular Ca^2+^ activate calmodulin and other kinases, which can phosphorylate and thereby activate NHE1 together with the initial NOX-mediated increase in intracellular H^+^ concentration [10,76,79,80,81]. The chemoattractant-induced overshooting alkalization is largely NHE1 dependent, as shown by NHE1 inhibition with the NHE1-specific inhibitor ((4-Cyanobenzo[b]thiophene-2-carbonyl)guanidine, methanesulfonate), while inhibition of the H^+^/K^+^-ATPase (by Omeprazole), H^+^-ATPase (by Bafilomycin), or the H_v_1 channel (by zinc) did not prevent C5a-induced alkalization significantly [10].

Following initial stimulation, this intracellular alkalization continues for up to an hour and modulates various crucial cellular functions, including fMLF-induced IL-8 release and C5a-induced increase in glucose metabolism [10,49]. This latter mechanism results in increased lactate generation, which is released in symport with H^+^ via monocarboxylate transporters, thereby contributing to extracellular lactate acidosis [10]. It is noteworthy that the alkalization of fMLF-stimulated neutrophils is increased by the presence of extracellular HCO_3_^−^ [82]. Additionally, the Cl^−^/HCO_3_^−^ exchanger (anion exchanger 1, AE1) appears to be inhibited by fMLF [82]. Accordingly, it was proposed that in general a sodium-proton exchange (likely NHE1) is responsible for recovering the intracellular pH from an acidic load, while a Cl^−^/HCO_3_^−^ exchange mechanism (likely AE1) appears to mediate the recovery from an alkaline load [82,83]. In this context, many previous studies of neutrophil alkalization must be interpreted with caution, because HCO_3_^−^ has frequently not been supplemented in culture media, presumably to facilitate the control of the extracellular pH. Regarding ion fluxes, changes in intracellular pH interact with CRAC-mediated Ca^2+^-influx and the release of O_2_^•−^. For example, intracellular alkalosis increases intracellular Ca^2+^ and thus cellular function, including migration [84,85]. Additionally, inhibition of fMLF-induced intracellular alkalization reduced but did not completely inhibit O_2_^•−^ release [49,79,86]. Besides the frequent lack of HCO_3_^−^ and other important blood ions and proteins in the buffer for neutrophil experiments, it is also noteworthy that most of the described mechanisms have been demonstrated mainly for fMLF and/or C5a stimulated neutrophils.

The discussed ion–water fluxes in neutrophils after stimulation with chemoattractants also interact closely with cellular migration, which has been addressed in other reviews [87,88,89]. Here, we only briefly summarize this, because changes of neutrophil cell size and shape are also impaired during sepsis as discussed below. Neutrophils stimulated with chemoattractants respond within minutes with a pronounced change in cellular shape towards an oval, elongated form as well as a moderate increase in cellular size [11,50,90]. In general, migration involves cellular polarization, including an influx of osmolytes such as Na^+^ as well as water at the protrusive end, active reorganization of the actin cytoskeleton, and an efflux of ions and water at the retractive pole [13,87,88,89]. Actin polymerization at the protruding cell pole is driven by Cdc42 activity, which depends on local intracellular alkalization by NHE1 and others [91,92]. Additionally, as demonstrated in fibroblast, NHE1 acts as an anchor for actin filaments [93]. In the context of neutrophil migration, the influx of water contributing to the increase in neutrophil cell size is regulated via Ras-related C3 botulinum toxin substrate 1 (Rac1)-dependent pathways phosphorylating and thus activating aquaporin (AQP)9 [94,95,96,97]. Of note, this process starts within minutes of neutrophil stimulation with fMLF and correlates temporally with actin polymerization [97]. Additionally, AQP1 is involved in neutrophil water flux, as discussed below [98].

## 5. Sepsis-Induced Neutrophil Dysfunction and Its Correlation with Ion and Water Flux

In sepsis, neutrophils are exposed to a broad spectrum of inflammatory mediators, including cytokines, chemokines, and complement fragments as well as to bacterial endotoxins, which can possibly impact neutrophil ion and water flux and thereby modulate neutrophilic functions. In accordance with this, numerous pivotal functions of neutrophils, including phagocytosis, oxidative burst production, and chemotactic activity, can be impaired during severe sepsis [99,100,101].

In agreement with the proposed alterations of ion transport proteins, an increase in neutrophil size and cell volume can be observed in murine sepsis [8,11] as well as an elevated intracellular pH in neutrophils from patients with sepsis [10]. Moreover, NHE1 inhibition with amiloride markedly reduced LPS-induced lung neutrophil infiltration in murine sepsis, indicating the requirement of NHE1 (or other Na^+^ transport proteins) for neutrophil chemotaxis in sepsis [15]. In this in-vivo study, it was not clearly distinguishable whether the effect could be attributed to the inhibition of neutrophil NHE1 or reduced production of macrophage- and epithelium-derived cytokines and chemoattractants, because NHE1 is ubiquitously expressed in the lung tissue. However, in-vitro studies could demonstrate that NHE1-dependent regulation of intracellular pH is involved in chemotaxis of human neutrophils under inflammatory conditions [42,102]. In general, targeting ion transport proteins such as NHE1 during sepsis might be an interesting clinical option in systemic inflammation [9] or for patients with cancer [103]. However, to our knowledge, this has not been translated to patients with sepsis.

One consequence of this “metabolic switch” when turned on during sepsis may also feature shape changes of neutrophils reflected by an increase in the cellular length/width ratio [11]. Of note, the in-vivo morphological changes of neutrophils during sepsis were associated with a slight increase in their volume [11]. Other mechanisms may be involved for sufficient neutrophil functions, such as members of the transient potential receptor (TRP) channel family which control the Ca^2+^ currents and thereby modulate any micro-movement efforts, including during migration, chemotaxis, phagocytosis, and cytokine release [13].

Controlling Ca^2+^ levels in neutrophils may also constitute a valuable strategy to modulate neutrophil-drive inflammation and/or to restore neutrophil function [104,105]. For example, in patients with uncontrolled noninsulin-dependent diabetes mellitus, an increase in basal Ca^2+^ was associated with an impairment on phagocytotic activity, which could be ameliorated with the calcium channel blocker amlodipine [106]. Likewise, an increase in intracellular Ca^2+^ is linked with reduced bacterial clearance of pathogens in neutrophils from individuals with cystic fibrosis, which also was improved by reducing intracellular Ca^2+^ [107]. Further studies need to elucidate basal Ca^2+^ levels and to explore the possibility to target Ca^2+^ homeostasis as mediator of neutrophil dysfunction during sepsis.

In addition to ion transport proteins, AQPs modulate fluid shifts in neutrophils, albeit their specific function in sepsis remains rather speculative. AQP5 protein expression has been reported to be downregulated during inflammation as a response to proinflammatory cytokines [108,109]. Interestingly, modulation of AQP5 expression is associated with altered neutrophil function, and there is increasing evidence that the AQP5 expression level in immune cells impacts survival rate in sepsis patients, proposing AQP5 as an interesting immune-modulatory therapeutic target. For example, high AQP5 mRNA expression in the blood of patients with sepsis was related to a higher mortality rate that was associated with greater AQP promotor methylation at a putative nuclear factor kappa-light-chain-enhancer of activated B cells (NF-κB) binding site [110]. Remarkably, genetic knock out or loss-of-function single-nucleotide polymorphism in the AQP5 gene promotor resulted in experimental and clinical improvement of sepsis and adult respiratory distress syndrome (ARDS) outcome [111,112]. However, the relationship between AQP5 and neutrophils or neutrophil-mediated inflammation remains to be elucidated. AQP9 has been reported to be increased during systemic inflammation [97], which could contribute to morphological changes and functional impairment of neutrophils during sepsis. In addition, elevated AQP1 expression was observed in leukocytes of patients with sepsis [98]. Though the latter was accompanied by an increase in mercury sensitive membrane water permeability, the specific biologic function and impact on neutrophil function needs to be determined further. In macrophages, AQP1 has been shown to promote cell migration [113]. Therefore, sepsis-induced AQP1 upregulation may impact neutrophil migration and chemotaxis. Moreover, because AQP1 does not only provide a water pore but also facilitates bicarbonate diffusion across biological membranes as concluded by atomistic molecular dynamics simulations, modulatory effects on intracellular pH adjustment appear possible [114].

**Figure 2:**
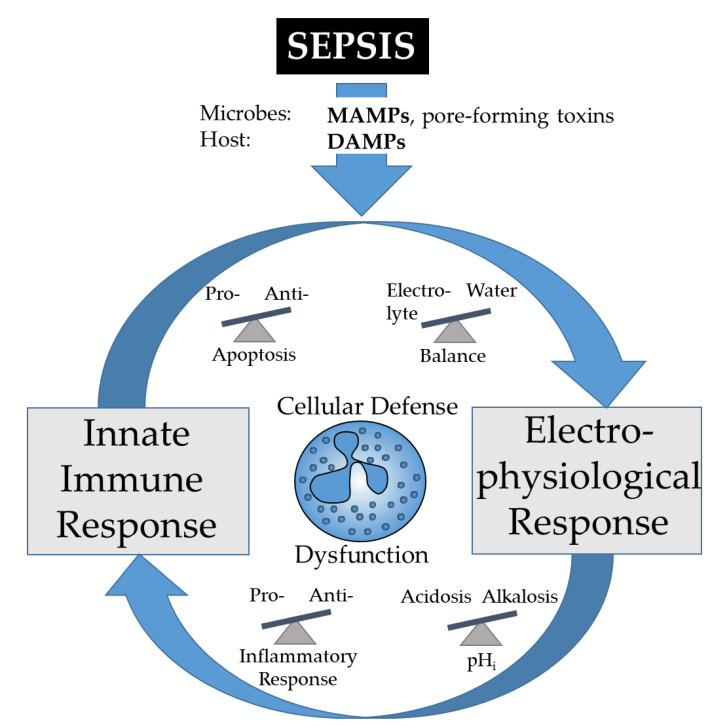
During sepsis, microbe-associated molecular patterns (MAMPs) and damage-associated molecular patterns (DAMPs) may alter various homeostatic balances. The resulting electro-physiological changes including alterations of the intracellular pH (pH_i_) will affect the inflammatory response of innate immunity and in consequence, vital cell functions. The mounted immune response including the feed-in of MAMPs and DAMPs will further affect cellular electrophysiology, which finally may form a vicious circle resulting in cellular dysfunction and death.

Changes in neutrophil ion and water homeostasis, frequently measured by changes in cell size, have been of increasing interest as diagnostic and prognostic markers in sepsis and other systemic inflammation conditions. A meta-analysis of the Area Under the Receiver Operating Characteristic Curve (AUROC) reported that a mean neutrophil volume of 0.87 (0.83–0.89) was an indicator for sepsis [12]. In accordance, alterations in neutrophil cell size occurred prior to organ dysfunction after severe injuries [16]. However, we are only beginning to understand the coherences between the ion–water regulation of neutrophils and their functions. Particularly during sepsis, the immune response and pathophysiological reaction may result not only in an immune suppression, but also in an altered ion–water homeostasis and dysfunctional cellular barriers [14]. Conversely, inflammation-driven alterations of the ion–water equilibrium may result in insufficient organ and immune responses, creating a vicious pathophysiological circle (Figure 2).

### 5.1. Alteration of the Membrane Integrity by MAMPs and DAMPs

Some MAMPs and DAMPs can disrupt the integrity of the leukocyte membrane by pore-forming molecules. In the case of MAMPs, various bacteria-derived toxins can generate differently sized pores in neutrophils which in turn lead to ion and water imbalances and in the worst case to cell death. Some sepsis-relevant microbial toxins are exemplarily summarized: Panton-Valentine leukocidin (PVL) is a toxin produced by some staphylococcal strains which can interact with the C5aR1 and induce lytic pores in neutrophils [115] as well as NETosis [116]. An important virulence factor for pseudomonas aeruginosa is the exolysin A (ExlA) which punctures holes into the plasma membranes and activates the inflammasome [117]. Furthermore, Escherichia. coli with its toxin α-hemolysin have been reported to form detrimental pores in neutrophils [118]. Last, Pneumolysin (Ply), an important virulence factor of Streptococcus pneumoniae, triggers neutrophil activation including Ca^2+^ influx, K^+^ efflux, activation of NF-κB as well as synthesis of IL-1β and IL-8 [119,120]. Overall, membrane-targeting immune evasion strategies are important drivers of septic courses. Of note, neutrophils can become resistant against some cytolysins by reprogramming the membrane cholesterol composition [121].

Important representatives for DAMPs are histones and extracellular ATP. For example, histone H4 causes neutrophil membrane depolarization, a rise in intracellular Ca^2+^ and the release of myeloperoxidase and IL-8 [122]. Interestingly, histone-induced cell death of neutrophils is ameliorated in the presence of fibrinogen, providing a potential therapeutic rationale reducing the detrimental effects of histones on neutrophils by preventing fibrinogen depletion during sepsis [123]. ATP, which can reach locally high concentrations, activates purinergic receptors, including the purinergic P2X7 receptor (P2X7R), which alters the membrane potential, induces cellular signaling and mounts an oxidative burst [124]. At higher concentrations, ATP-induced activation of P2X7R can lead to formation of macro-pores (with a molecular passage up to 900 Da) and thus permeabilization of neutrophils including subsequent cellular swelling [125]. In pyroptosis and septic conditions, ATP seems to contribute to the loss of membrane integrity by pore formation and the pathophysiological consequences [126]. However, it needs to be stated, that data on P2X7R expression and function in neutrophils are ambiguous. While many studies found expression of P2X7R in neutrophils [125,127,128,129,130], others reported an absence of P2X7R [131,132,133]. Whether these inconsistencies result from differences in research protocols or account for subpopulations of neutrophils remains to be answered [37].

### 5.2. Changes of Phagocytotic Activity

SOCE in neutrophils, regulated by the Ca^2+^ sensors STIM1 and 2, which activate the Ca^2+^ channel Orai1, guarantees sustained Ca^2+^-influx when activated (e.g., by MAMPs, STIM1) and thereby generates intracellular “Ca^2+^ hotspots” which augment phagocytosis [134] and promote reactive oxygen species (ROS) generation during inflammatory responses [52,57,135,136].

Contradictory reports have been published regarding the phagocytic and oxidative burst activity of neutrophils during sepsis, which, however, appear mainly dependent on the timing and severity of sepsis, the maturity of neutrophils, and the local environment [137,138]. During the early phases of sepsis, for example, the first 48 h after diagnosis, phagocytic activity of neutrophils has been described to be enhanced [139] and during advanced stages of sepsis as impaired if not defective [5,140]. One mechanism for sepsis-caused impairment of phagocytic activity involves enhanced levels of C5a, which leads to inhibition of RhoA activation and thus prevents actin polymerization and phagocytic processes [41]. Of note, neutrophils within the bloodstream exhibit minimal phagocytic activity but rapidly develop this function during their emigration into infected tissue [141]. Therefore, it is likely that ion transport proteins are crucially involved in acquiring the phagocytic activity. Indeed, neutrophils lacking the anion channel ClC-3 exhibit a reduced phagocytotic and NOX activity [142]. Furthermore, expression of the cAMP-activated chloride channel (CFTR) has been demonstrated in neutrophils on mRNA and protein level, although at rather low levels [143,144]. Involvement of CFTR in bacterial killing activity of neutrophils via its contribution to the generation of hypochlorite (HOCl) in the phagolysosome has been discussed [144,145,146,147]. However, the exact underlying mechanisms need further investigation.

### 5.3. Reduced Oxidative Burst

To kill phagocytized bacteria, effective antimicrobial mechanisms are essential. These mainly involve an oxidative burst with reactive oxygen species (ROS) generation that can be oxygen-dependent or -independent [141]. Oxygen-required ROS production mainly occurs by the NOX complex [148]. For example, upon TLR or C5aR stimulation by microorganisms and the MAMP-detecting complement system, respectively, intracellular signaling leads to phosphorylation and translocation of the usually dormant intracellular key enzyme p47^phox^ to the membrane, where it assembles with other components, including p40^phox^, p67^phox^, Rac1/2, and flavocytochrome b558, to the NOX complex [149]. The electron shift within the NOX reduces oxygen to form superoxide anions (O_2_^•−^), which is subjected to dismutation generating hydrogen peroxide (H_2_O_2_) and ultimately HOCl via the myeloperoxidase [149,150]. The electron transport requires the efflux of protons or Cl^−^-conductance for compensation of the electrical charge out of the vacuole [64,151,152,153]. Of note, during severe sepsis, together with excessive generation of C5a, the NOX activity is impaired if not abolished. The underlying mechanisms appear to be a C5a-induced inhibition of the phosphorylation and translocation of the key enzyme p47^phox^ and impaired ROS generation [5,41,154], which, in summary, results in an impaired bacterial clearance during severe sepsis. In this context, an impairment of the C5a-induced depolarization in neutrophils has been demonstrated in systemic inflammation after porcine hemorrhagic shock [48] as well as after exposing neutrophils from healthy volunteers to serum from patients with trauma and/or patients with trauma-related sepsis [155], but not to date in non-trauma-related sepsis. By contrast, in-vitro pretreatment with LPS 50 ng/ml for one hour enhanced fMLF-induced ROS generation, possibly by elevating the intracellular Ca^2+^ levels of resting neutrophils [45], which could be explained by a temporal component, for example, an initial pronounced ROS generation and a subsequent functional impairment in prolonged inflammation. Intriguingly, while resting neutrophils have a normally-distributed membrane potential as assessed by various fluorescent dyes [45,48], LPS-pretreatment induced a bimodal heterogeneity of resting membrane potential that was partially unified by fMLF stimulation [45].

Moreover, extracellular acidification, as present in local and systemic inflammation, directly inhibits bacterial killing by neutrophils, while in contrast promoting phagocytosis [43]. In accordance, intracellular acidification by short-chain fatty acids, metabolic by-products of Bacteroides species, inhibited the respiratory burst of neutrophils [156]. By contrast, in-vitro extracellular acidosis enhanced C5a-induced depolarization [48]; this discrepancy needs further research.

Regarding the oxygen-independent mechanism of bacterial killing, lysosomal enzymes are released into the phagosomes, where after an initial alkalization phase the now phagolysosomes become acidified by activation of the Na^+^/H^+^ exchanger [157]. In addition to NHE1, the murine H^+^ channel VSOP and its respective human homologue H_v_1 are considered essential for the excretion of protons produced by NOX [64,69]. The finding that neutrophils from H_v_1^−^/^−^ mice had a substantially reduced superoxide production and impaired bacterial clearance both in-vitro and in-vivo indicates the requirement of H_v_1 for sufficient antibacterial activity in sepsis [158]. Notably, although the discussed ion transport proteins and mechanisms of intracellular pH regulation are indisputably essential for the key functions of neutrophils, the question of whether direct alterations of, for example, NHE1 or VSOP/H_v_1 expression or activity actually do contribute to neutrophil dysfunction in severe sepsis has to date hardly been addressed and requires further research.

Moreover, Ca^2+^-homeostasis in neutrophils also appears crucially involved in the mounting of an oxidative burst and other antimicrobial functions. Several Ca^2+^-conductive ion channels are expressed in neutrophils, including the TRP family and store-operated Ca^2+^ channels. TRPM2, a Ca^2+^ permeable, non-selective cation channel, which is activated by ADPr, temperature, oxidative stress, and Ca^2+^ [159], has been proposed to play important roles in modulating Ca^2+^ mobilization and oxidative stress in neutrophils. For example, during experimental sepsis in mice infected with Listeria monocytogenes, the TRPM2 cation channel modulated membrane depolarization, Ca^2+^ mobilization, and subsequent ROS generation [160]. In this study, a lack of TRPM2 resulted in reduced sepsis survival, and in neutrophils led to enhanced depolarization, dysregulation of intracellular Ca^2+^, and aggravated oxidative burst, all of which might be harmful to the host [160]. Another mechanism involves the ATP-gated purinoceptor P2X1 ion channel, which with its relatively high Ca^2+^ permeability could limit the oxidative burst response during LPS-induced murine sepsis [161]. In this context, it is also noteworthy that ROS exhibit not only local antimicrobial functions but also can act as signaling molecules for neutrophils in an autocrine manner. In turn, this can further regulate and drive the inflammatory response of neutrophils in conditions like sepsis [49,162].

Finally, ion concentrations and pH values of the local inflammatory microenvironment of the neutrophils will self-evidently to some extent alter the intracellular milieu and thereby also influence the phagocytic and oxidative burst activity [78,163,164]. However, this has barely been studied in-vivo at the local site of inflammation during sepsis.

## 6. Cell Death during Inflammation and Sepsis

A multitude of triggers and signaling pathways have been described in initiating and regulating neutrophil death [126,165]. Rapid generation and secretion of proinflammatory mediators, toxic granular substances, or NETs by neutrophils require lytic cell death mechanisms [165]. Neutrophil death pathways involving cell membrane lysis comprise a fail-safe mechanism designed to eradicate intracellular pathogens that escape pathogen-induced cell death-mediated anti-inflammatory pathways [166]. In sepsis, cell death is modulated by certain conditions, for example, a delay of neutrophil apoptosis by extracellular acidosis or stimulation with C5a [43,167]. By contrast, another study reported that exposure to Escherichia coli at low pathogen-to-neutrophil ratios resulted in intracellular alkalization and inhibited apoptosis and vice versa [163]. In addition to apoptosis (less inflammatory), neutrophils may undergo necroptosis, pyroptosis (both proinflammatory), or NETosis, as reviewed by others [126,165]. Here, we will mainly focus on the role of membrane pores and ion transport proteins in pyroptosis and NETosis.

### 6.1. Pyroptosis

Pyroptosis is activated by cytosolic inflammasome perturbations. Inflammasome activation can be induced by intracellular pathogens or by intracellular exposure to LPS that result in activation of inflammatory caspases that cleave gasdermin D (GSDMD) [140,165]. Interestingly, pyroptosis appears to be restricted to activation of the non-canonical inflammasome but not the classical inflammasome, which induces caspase-1 activation [168]. Caspase-1 activation only results in the generation of sublytic pores to enable IL-1β secretion without concomitant cell lysis [168].

Recent findings documented that GSDMD can also be processed by neutrophil elastase. Cleavage occurs upstream of the canonical caspase cleavage site, but still produces pore-forming *N*-terminal fragments [169,170]. However, the functional effects are less clear. It was proposed that GSDMD cleavage results in the formation of lytic pores and spontaneous neutrophil death, establishing GSDMD as a negative regulator of innate immunity [169]. By contrast, GSDMD activation by neutrophil elastase was found to drive cell lysis and NET extrusion, an important antimicrobial defense mechanism [168]. Therefore, it appears that GSDMD activity in neutrophils can either promote or dampen the host defense [171].

Extracellular ATP can activate inflammasome signaling in neutrophils via activation of P2X7Rs on neutrophils [172]. P2X7R is a non-selective cation channel that mediates rapid Ca^2+^- and Na^+^-influx and K^+^-efflux, which results in NLRP3 inflammasome activation [129,173]. Whether this is independent from inflammasome activation by intracellular pathogens or an amplification mechanism needs to be determined. At least in macrophages, cytosolic LPS stimulation-induced caspase-11-dependent cleavage of pannexin-1 resulted in ATP release via pannexin hemichannels, which in turn activated P2X7Rs. In the absence of P2X7 or pannexin-1, cytosolic LPS-induced pyroptosis was abrogated [174].

### 6.2. NETosis

Activated neutrophils can neutralize invaders by releasing NETs [175] in a process termed NETosis. NETs are webs of neutrophil DNA coated with histones and antimicrobial proteins [176]. Initially described as a means for neutrophils to neutralize pathogens, NET release also occurs in sterile inflammation, promotes thrombosis, and can mediate tissue damage [177]. NETosis involves several distinct and sequential morphological changes in the neutrophil [176]. Chromatin needs to be decondensed and released from the nucleus and the cytoskeleton, organelles, and intracellular, nuclear, and plasma membranes must be remodeled [177]. NETs are extruded in host defense through either lytic (suicidal and noncanonical NETosis) or vital NETosis. Although all of these ultimately lead to neutrophil death, NETosis is a distinct cellular program from apoptosis and necroptosis, and true NET structures are not usually generated during these latter forms of cell death [176,177].

Increases in intracellular Ca^2+^ mediates ROS production, essential for lytic NETosis. This is probably a result of NOX activation [178,179], activation of small conductance calcium-activated potassium channel (SK) member SK3, and production of mitochondrial ROS [180]. In agreement with this, BAPTA-AM, a potent intracellular Ca^2+^ chelator, inhibited NETosis [181]. However, it remains unclear whether the elevation of cytoplasmic Ca^2+^ levels originates from Ca^2+^ entry [178] or release from intracellular Ca^2+^ stores [180]. Depending on the experimental conditions, the use of extracellular Ca^2+^ chelators over a prolonged period could induce a homeostatic response in which a decrease in extracellular Ca^2+^ results in increased Ca^2+^ release by the ER to maintain intracellular Ca^2+^. Such a phenomenon could explain, in part, such a discrepancy between the studies [182]. Activation of the TRPM2 channel reduced the NET formation, and cell death in neutrophils in a model of Listeria monocytogenes infection [160]. Interestingly, a HCO_3_^−^-mediated alkalization of neutrophils favored spontaneous and LPS-induced NETosis, possibly because of modulation of intracellular Ca^2+^ [84].

In lytic (suicidal and noncanonical) NETosis, NET extrusion occurs via GSDMD pores [168,170] and/or as a result of entropic chromatin swelling [183] that ultimately induce plasma membrane rupture to enable NET release. More recently, another type of NETosis has been identified and termed vital NETosis. In vital NETosis, NETs are rapidly released from neutrophils via exocytosis. Vital NETosis does not immediately induce cell death and neutrophils can still perform cellular functions (e.g., migration) [176,184,185]. It is readily conceivable that vital NETosis is thus also dependent on Ca^2+^ signals that themselves depend on intracellular or plasma membrane Ca^2+^ channels.

### 6.3. Cellular Death Converges in GSDMD

Apoptosis, pyroptosis, and NETosis were traditionally considered to be independent of one another, but emerging evidence indicates that there is extensive cross-talk between them. All three pathways can converge upon the activation of the same cell death effector—the pore-forming protein GSDMD [171]. Similar to pyroptosis and NETosis, GSDMD activation has also been related to apoptosis. Activation of apoptotic caspase-8 can either directly result in GSDMD cleavage [186] or indirectly induce activation of plasma membrane channels [187], K^+^ efflux, and NLRP3 inflammasome assembly, which result in NLRP3-dependent caspase-1 activation and GSDMD cleavage [171].

GSDMD consists of a pore-forming *N*-terminal domain (GSDMD-N) connected via a linker to the regulatory C-terminal domain. Cleavage of GSDMD at a conserved site releases GSDMD-N from the auto-inhibitory C-domain. GSDMD-N has the ability to translocate to the inner leaflet of the plasma membrane, where it oligomerizes and induces the opening of membrane pores [171,188,189,190,191,192,193]. The pores constitute non-selective membrane channels that allow the passage of ions, cytokines, and other small cytosolic molecules [194]. The resulting shift in cytoplasmic ion concentrations (in particular the large influx of Na^+^) causes an increase in cell volume due to water influx and can result in osmotic cell lysis. This likely depends on the number of GSDMD pores in the plasma membrane. The number of GSDMD pores can be limited by a recently discovered mechanism that actively removes GSDMD pores from the plasma membrane. Ca^2+^-influx via GSDMD pores recruits endosomal sorting complexes required for transport (ESCRT) to damaged areas of the plasma membrane to remove GSDMD pores and to maintain membrane integrity [195]. This is likely a rapid mechanism, curtailing osmotic swelling at an early stage. If few GSDMD pores are present, the cell can react by initiating compensatory mechanisms to decrease volume, called regulatory volume decrease (RVD). Among these are swelling-activated K^+^, Cl^−^, and organic osmolyte (e.g., taurine) channels that export these solutes and their accompanying water [194]. In particular, neutrophils express volume-regulated anion channels (VRACs). These are sensitive to shifts in cytoplasmic ionic strength and could constitute such a RVD mechanism [196,197]. A detailed understanding of the specific RVD mechanisms and their contribution to regulate apoptosis, pyroptosis, and NET-osis in neutrophils, however, is still elusive. If GSDMD pores are present in high numbers, exceeding the cell’s compensatory capabilities, cell volume inevitably increases. Once the volume exceeds membrane capacity, this results in membrane rupture and cell lysis.

## 7. Conclusions and Outlook

Ion and water fluxes regulate essential neutrophil functions. Their alterations during sepsis impair vital cellular functions and may thereby contribute to immune and organ dysfunctions. Fields of particular interest include first, a further elucidation of the ion–water disbalance upon excessive neutrophil activation, second, an in-depth evaluation of physiological parameters, including intracellular pH and cellular size of neutrophils as a diagnostic and/or prognostic marker of immune dysfunction, and third, a meaningful translation to the bedside by modulating ion–water based alterations, for example, by pharmacological targeting of perpetrator ion transport proteins, such as NHE1, during sepsis.

## Data Availability

No new data were created or analyzed in this study. Data sharing is not applicable to this article.

## Abbreviations

ADPr	ADP-ribose
AE1	anion exchanger 1
AQP	Aquaporin
ARDS	adult respiratory distress syndrome
ATP	adenosine triphosphate
AUROC	Area Under the Receiver Operating Characteristic Curve
C5a	complement factor 5a
C5aR1	C5a receptor 1
cADPr	cyclic ADPr
CaM	calmodulin
CFTR	cAMP-activated chloride channel
Cdc42	cell division control protein 42
CLIC	chloride intracellular channels
CR	complement receptor
CRAC	Ca^2+^ release-activated Ca^2+^
DAG	diacylglycerol
DAMP	damage-associated molecular pattern
ER	endoplasmatic reticulum
ESCRT	endosomal sorting complexes required for transport
ExlA	exolysin A
fMIVIL	*N*-formyl-Met-Ile-Val-Ile-Leu
fMLF	*N*-formyl-methionyl-leucyl-phenylalanine
FRP	formyl-peptide receptor
Glu	glucose
GLUT1	glucose transporter 1
GPCR	G protein-coupled receptors
GSDMD	gasdermin D
HMGB1	high mobility group box 1
H_v_1	voltage-sensing domain only protein
IL	interleukin
IP_3_	inositol 1,4,5-trisphosphate
LTB_4_	leukotriene B_4_
MAMP	microbial-associated molecular pattern
MCT	monocarboxylate transporter
NAD^+^	nicotinamide adenine dinucleotide
NADPH	nicotinamide adenine dinucleotide phosphate
NET	neutrophil extracellular trap
NF-κB	nuclear factor kappa-light-chain-enhancer of activated B cells
NHE1	sodium-proton-exchanger 1
NLR	NOD-like receptor
NLRP3	NOD-like receptor family pyrin domain containing 3
NOD	nucleotide-binding oligomerization domain
NOX	NADPH oxidase
P2X7R	purinergic P2X7 receptor
p47^phox^	p47 phagocyte oxidase
PAF	platelet-activating factor
PIP_2_	phosphatidylinositol 4,5-bisphosphate
PKC	protein kinase C
Ply	pneumolysin
PRP	pattern recognition receptor
PVL	Panton-Valentine leukocidin
RAC1	Ras-related C3 botulinum toxin substrate 1
RAGE	receptor for advanced glycation end products
ROS	reactive oxygen species
RVD	regulatory volume decrease
SK	small conductance calcium-activated potassium channels
SOCE	store-operated calcium entry
STIM	stromal interaction molecule
TLR	toll-like receptor
TRP	transient potential receptor
TRPM	transient receptor potential melastatin
VRAC	volume-regulated anion channel
VSOP	voltage sensing domain only protein
